# Dietary insulin load and insulin index are associated with the risk of insulin resistance: a prospective approach in tehran lipid and glucose study

**DOI:** 10.1186/s40200-016-0247-5

**Published:** 2016-07-20

**Authors:** Parvin Mirmiran, Saeed Esfandiari, Zahra Bahadoran, Maryam Tohidi, Fereidoun Azizi

**Affiliations:** 1 Nutrition and Endocrine Research Center, and Obesity Research Center, Research Institute for Endocrine Sciences, Shahid Beheshti University of Medical Sciences, Tehran, Iran; 2 Prevention of Metabolic Disorders Research Center, Research Institute for Endocrine Sciences, Shahid Beheshti University of Medical Sciences, Tehran, Iran; 3 Endocrine Research Center, Research Institute for Endocrine Sciences, Shahid Beheshti University of Medical Sciences, Tehran, Iran; 4 No. 24., Shahid Erabi St., Yeman St., P.O. Box: 19395-4763, Velenjak, Tehran Iran

**Keywords:** Dietary insulin index, Dietary insulin load, Insulin resistance

## Abstract

**Background:**

The aim of this study was to investigate the relationship between dietary insulin load (DIL) and insulin index (DII) and the risk of insulin resistance in Tehranian adults.

**Methods:**

In this study, 927 men and women, aged 22–80 years, participated in Tehran Lipid and Glucose Study were included. Fasting serum insulin and glucose were measured at baseline and again after a 3-year of follow-up. Usual dietary intakes were measured using a validated 168 item semi-quantitative food frequency questionnaire and DIL and DII were calculated. Logistic regression models were used to estimate the occurrence of the IR across tertile categories of DIL and DII with adjustment for potential confounding variables.

**Result:**

Mean age of participants was 40.71 ± 12.14 y, and mean body mass index (BMI) was 27.23 ± 4.9 kg/m^2^, at baseline. Mean of DIL and DII was 937 ± 254 and 84.0 ± 6.3. Participants with higher DIL had higher weight and waist circumference at baseline (*P* < 0.05). A borderline positive association was observed between DII and the risk of insulin resistance in fully adjusted model (odds ratio = 1.66, 95 % confidence interval = 0.96–2.86, *P* for trend = 0.06). After adjustment of potential confounders, highest compared to the lowest tertile of DIL was also significantly associated with increased risk of insulin resistance (odds ratio = 1.69, 95 % confidence interval = 1.01–2.89, *P* for trend = 0.06).

**Conclusion:**

Dietary insulin load and DII could be considered as independent dietary risk factors for development of insulin resistance.

## Background

Insulin resistance, defined as a state of reduced insulin secretion or inability of insulin to optimally stimulate transport of glucose into the peripheral tissues, is a major player in the pathogenesis of the metabolic syndrome and type 2 diabetes [1, 2].

Although the exact causes of insulin resistance are not completely understood, but insulin resistance is related to metabolic disorders that are underlying causes of major chronic diseases; current evidence highlight that diet has a determinant role in the pathogenesis of insulin resistance and related metabolic disorders such as metabolic syndrome and type 2 diabetes [3, 4]. Studies suggest that dietary habits, regular dietary pattern, macronutrient such as carbohydrate, fiber, fats and micronutrients may be related to incidence of insulin resistance [5–8].

Foods ability to induce postprandial insulin secretion may be relevant in the context of the prevention and management of insulin resistance and type 2 diabetes [9]; although glycemic index of foods provide useful information regarding glycemic response of foods based on their carbohydrate content, it cannot provide a guide to relative insulin response of a large majority of food items [10]. A dietary insulin index (DII) has been defined as a direct index of the postprandial insulin response to a test food in comparison with an isoenergetic portion of a reference food (analogous to the glycemic index, either glucose or white bread). Dietary insulin index is more suitable than glycemic index (GI) for exploring a relationship with development of chronic disease because of insulin index is directly based on insulin response. Based on DII we can define dietary insulin load (DIL) by multiplying the DII of each food by its energy content and the consumption frequency.

Holt et al. for the first time, systematically compared postprandial insulin responses to isoenergetic portions of some common foods and developed an insulin score for each food on the basis of its insulinemic effect in relation to a reference food [11]. Dietary insulin index depends on carbohydrate, quantity and quality of protein, fat and their interactions; it is a novel algorithm of ranking foods based on the insulin response and also a simple and practical measure in nutritional epidemiology to evaluate effect of diet on insulin hemostasis and development of type 2 diabetes [11, 12]. Findings from epidemiological studies indicate that DIL and DII could be related to some metabolic disorders including increased inflammatory cytokines, elevated atherogenic lipid profiles and non-communicable chronic diseases [9, 13–16].

In this study, we aimed to investigate the association of DIL and DII and the risk of insulin resistance in a 3-year follow-up in the framework of Tehran Lipid and Glucose Study.

## Methods

### Study population

This study was conducted within the framework of the Tehran Lipid and Glucose Study (TLGS), an ongoing community-based prospective study being conducted to investigate and prevent non-communicable diseases, in a representative sample in district 13 of Tehran, the capital city of Iran [17]. The current study was conducted on adult men and women with complete data (demographics, anthropometrics, biochemical and dietary data), participated in the third (2006–2008) and fourth (2009–2011) TLGS examinations. Participants were excluded from the final analysis if they had unexplained energy intake (<800 kcal/d or >4200 kcal/d) or were on specific diets (*n* = 262), had no follow-up information on anthropometrics and biochemical measurements at the second examination (2009–2011). After exclusion of the participants with insulin resistance at baseline, finally, data of 927 participants was included in the analysis. The mean duration of the follow-up was approximately 3 years.

### Demographic and anthropometric measures

Demographics, anthropometrics, and biochemical measures were evaluated at baseline and follow-up examination, after 3-yaers. Trained interviewers collected information using pretested questionnaires. Smoking status was obtained during face-to-face interviews. Physical activity level was assessed based on the frequency and time spent on light, moderate, high and very high intensity activities, according to the list of common activities of daily life over the past year. Physical activity levels were expressed as metabolic equivalent hours per week (METs h/week).

Weight was measured to the nearest 100 g using digital scales, while the subjects were minimally clothed, without shoes. Height was measured to the nearest 0.5 cm, in a standing position without shoes, using a tape meter. Body mass index was calculated as weight (kg) divided by square of the height (m^2^). Waist circumference (WC) was measured to the nearest 0.1 cm (at anatomical landmarks), at the widest portion, over light clothing, using a soft, tape meter, without any pressure to the body.

### Biochemical measures

Fasting blood samples were taken after 12–14 h, from all study participants at baseline and after 3-years of follow-up. Fasting serum insulin was determined by the electrochemiluminescence immunoasaay (ECLIA), using Roche Diagnostics kits and the Roche/Hitachi Cobas e-411analyzer (GmbH, Mannheim, Germany). The intra- and inter-assay coefficients of variation for insulin were 1.2 and 3.5 %, respectively. Fasting plasma glucose was measured by the enzymatic colorimetric method using glucose oxidase. Inter- and intra- assay coefficient of variation of glucose assays was < 5 %.

Homeostatic model assessment of insulin resistance (HOMA-IR) was calculated as follows: Fasting insulin (μU/mL) × fasting glucose (mmol/L)/22.5; this index has been developed as a simple, inexpensive, and validated alternative tools for assessment of insulin resistance in epidemiological studies [18–20]. In the present study, IR was defined as HOMA-IR ≥ 3.2 [21].

### Dietary assessment, dietary insulin index and insulin load calculation

A 168-item food frequency questionnaire (FFQ) was used to assess typical food intake at first examination over the previous year. Trained dietitians asked participants to designate their intake frequency for each food item, consumed during the past year on a daily, weekly, or monthly basis. Portion sizes of consumed foods reported in household measures were then converted to grams. Energy and nutrient content of foods and beverages were analyzed using US Department of Agriculture Food Composition Table (FCT) because the Iranian FCT is incomplete, and has limited data on nutrient content of raw foods and beverages [22]. Finally, dietary intakes of participants including dietary energy and energy density, macronutrients, micronutrients, and food groups were determined.

Dietary insulin index was calculated from previous published estimates by Brand-Miller [11]; briefly, DII refers to the incremental insulin area under the curve over 2 h in response to the consumption of a 1000-kJ portion of the test food divided by the area under the curve after ingestion of a 1000-kJ portion of the reference food [9, 11]. In this study we matched food items based on correlation between similar food item of our FFQ according to their content of energy, fiber, carbohydrate, protein and fat. In the current study, the average DIL during the past year was calculated from the FFQ by multiplying the DII of each food by its energy content and the consumption frequency and summing over all reported food items as follows

$$ \mathrm{Insulin}\ \mathrm{load}\ \mathrm{of}\ \mathrm{food}\kern0.5em ={\displaystyle {\displaystyle \sum \left[\mathrm{insulin}\ \mathrm{index}\ \mathrm{of}\ \mathrm{food}\kern0.5em  \times \mathrm{energy}\ \mathrm{content}\ \mathrm{of}\ \mathrm{food}\ \left(\mathrm{kcal}/\mathrm{serving}\right) \times \mathrm{frequency}\ \mathrm{of}\ \mathrm{consumption}\ \left(\mathrm{servings}\ \mathrm{of}\ \mathrm{food}/\mathrm{day}\right)\right]}} $$

After using DII value for calculating average insulin load wcuee calculated the average DII for overall food items by dividing DIL by the total energy intake as follows

$$ \mathrm{Isulin}\ \mathrm{index}\ \mathrm{of}\ \mathrm{food} = \mathrm{Dietary}\ \mathrm{insulin}\ \mathrm{load}/\left\{{\displaystyle \sum \left[\mathrm{energy}\ \mathrm{content}\ \mathrm{of}\ \mathrm{food}\left(\frac{\mathrm{kcal}}{\mathrm{serving}}\right)\times \mathrm{frequency}\ \mathrm{of}\ \mathrm{consumption}\left(\frac{\mathrm{serving}\ \mathrm{of}\ \mathrm{food}}{\mathrm{day}}\right)\right]}\right\} $$

### Statistical analyses

All statistical analyses were conducted using SPSS (Version 16.0; Chicago, IL), and *P* values < 0.05 was considered significant.

Dietary intake of carotenoids was adjusted for total energy intake, based on the residuals methods [23]. Dietary insulin load and DII were categorized into tertiles (≤794, 794–1097, ≥1097) and (<81.8, 81.8–86.5, ≥86.0). Due to lack of defined cut off point for DII and DIL, and low power and sample size of the study, DII and DIL were categorized according to tertile values.

Participant characteristics were compared across tertile categories of total carotenoids intakes, using the analysis of variance or chi-square test.

A univariate analyses was performed for potential confounding variables; variables with P_E_ < 0.2 in the univariate analyses were selected for the final multivariable models; P_E_ (*P* value for entry) determines which variables should be included in the multivariable model.

To estimate the risk of IR across tertiles of DIL and DII, multiple regression models were used. The potential confounding variables adjusted in the models were sex, age at baseline (y, continuous), BMI (kg/m^2^, continuous), smoking status (yes or no), physical activity (MET-h/week, continuous), anti-diabetic medications (yes/no), and total energy intakes (kcal/d). A linear trend test was performed considering each ordinal score variable as a continuous variable in the model. To assess the overall trends of odds ratios of IR across tertiles DIL and DII, the median of each tertile was used as a continuous variable in the logistic regression models.

## Results

Mean age of participants was 40.71 ± 12.14 y, and mean BMI was 27.23 ± 4.9 kg/m^2^, at baseline. Mean of DIL and DII was 937 ± 254 and 84.0 ± 6.3. Median dietary II was 84.96. Mean fasting serum insulin was 9.1 ± 5.6 and 9.9 ± 6.5 μU/mL at baseline and after 3-years, respectively. The incidence rate of insulin resistance was 12.8 % after 3-years.

Characteristics of the participants across tertiles of DIL and DII are compared in Table 1. Subjects in the last tertile of DIL were more likely to be men (*P* < 0.05). Participants with higher DIL had higher weight at baseline also after 3 years (*P* < 0.05); higher waist circumference was also observed in participants with higher DIL, at baseline (89.8 vs. 87.8 cm, *P* < 0.05). There was no significant difference in FBS, serum insulin and HOMA-IR across tertiles of DIL and DII.

**Table 1:** Characteristics of the participants across tertiles of dietary insulin load and insulin index

	Dietary insulin index				Dietary insulin load	
	Tertile1	Tertile2	Tertile3	Tertile1	Tertile2	Tertile3
	<81.6	81.6–86.3	≥86.3	<794	794–1097	≥1097
Age at baseline *(yr)*	40.7 ± 12.0	38.8 ± 12.1	41.0 ± 12.2	39.53 ± 11.54	40.60 ± 12.17	40.46 ± 12.66
Men *(%)*	39.4	45.1	51.0	31.3	44.2	60.0*
Smoking *(%)*	14.2	9.1	11.6	14.0	9.7	10.3
Weight *(kg)*						
At baseline	70.5 ± 11.9	71.1 ± 13.1	71.9 ± 13.5	70.1 ± 12.0	69.8 ± 12.7	73.6 ± 13.6*
After 3-years	72.2 ± 12.1	73.1 ± 12.8	73.6 ± 13.6	71.5 ± 11.8	72.0 ± 12.9	75.3 ± 13.4*
Waist circumference *(cm)*						
At baseline	87.4 ± 12.1	88.2 ± 12.4	89.0 ± 12.2	87.8 ± 11.9	87.0 ± 12.6	89.8 ± 12.0*
After 3-years	91.8 ± 11.3	92.5 ± 10.5	93.3 ± 11.1	92.4 ± 10.8	91.6 ± 11.4	93.6 ± 10.7
Fasting serum glucose *(mg/dL)*						
At baseline	87.3 ± 11.9	86.8 ± 13.7	87.9 ± 15.2	86.3 ± 12.2	87.3 ± 13.4	88.5 ± 15.1
After 3-years	96.3 ± 21.1	94.9 ± 16.9	95.7 ± 19.1	93.9 ± 14.3	95.9 ± 20.9	97.1 ± 21.0
Fasting serum insulin *(mU/L)*						
At baseline	7.5 ± 2.9	7.6 ± 3.1	7.6 ± 3.1	7.6 ± 2.9	7.6 ± 3.2	7.5 ± 2.9
After 3-years	8.3 ± 3.9	8.8 ± 4.2	8.7 ± 4.4	8.4 ± 4.0	8.8 ± 4.4	8.6 ± 4.1
HOMA-IR						
At baseline	1.62 ± .67	1.63 ± .67	1.63 ± .68	1.63 ± .65	1.63 ± .70	1.62 ± .66
*After 3-years*	1.98 ± 1.10	2.11 ± 1.32	2.10 ± 1.18	2.05 ± 1.24	2.10 ± 1.18	2.04 ± 1.20

Data are mean ± SD (unless stated otherwise) * *P* < 0.05 (analysis of variance or chi-square test was used)

Dietary intakes of the participants across tertiles of dietary insulin load and insulin index is shown in Table 2. Total energy intake was significantly higher in the highest compared to the lowest quartile categories of DIL (2911 vs. 1650 kcal/d, *P* < 0.05). Participants with higher DIL and DII had also higher intakes of energy density, carbohydrates, dietary fiber and lower intakes of dietary fat (*P* < 0.05). Dietary intakes of fruits and vegetables decreased across increasing trend of DIL and DII (*P* < 0.05); participants who had higher dietary DIL had also lower intakes of whole grains and legumes (*P* < 0.05).

**Table 2:** Dietary intakes of the participants across tertiles of dietary insulin load and insulin index

	Dietary insulin index				Dietary insulin load	
	Tertile1	Tertile2	Tertile3	Tertile1	Tertile2	Tertile3
	< 81.6	81.6–86.3	≥86.3	<794	794–1097	≥1097
Energy *(kcal/d)*	2210 ± 680	2276 ± 715	2321 ± 734	1658 ± 414	2236 ± 439	2911 ± 604*
Energy density *(kcal/100 g of foods)*	91. 8 ± 19.2	96.0 ± 20.9	103 ± 24.9*	91.9 ± 20.6	94.0 ± 22.15	104 ± 21.0*
Carbohydrate *(% of energy)*	56.2 ± 7.1	57. 9 ± 7.5	58.7 ± 6.8*	55.3 ± 7.3	57.4 ± 7.1	60.1 ± 6.4*
Protein *(% of energy)*	13.9 ± 2. 6	13.4 ± 2.4	13. 7 ± 2.3	13.8 ± 2.7	13.5 ± 2.4	13.8 ± 2.2
Fat (% of energy)	32.4 ± 6.8	31.3 ± 7.5	30.2 ± 6. 7*	33.5 ± 7.3	31.8 ± 6.7	28.5 ± 6.1*
Fiber *(g/d)*	32.9 ± 0.9	35.7 ± 1.2	46.2 ± 1.8*	31.3 ± 1.1	36.9 ± 1.2	46.5 ± 1. 8*
Fruits *(g/d)*	424 ± 16.1	398 ± 16.1	350 ± 16.1*	438 ± 16.1	427 ± 16.1	306 ± 15.7*
Vegetables *(g/d)*	330 ± 10.7	292.0 ± 10.7	286 ± 10.7*	350 ± 10.7	350 ± 10.8	353 ± 10.6*
Whole grains *(g/d)*	22.7 ± 1.4	20.9 ± 1.4	19.4 ± 1.4	24.1 ± 1.4	21.8 ± 1.4	17.0 ± 1.4*
Legumes *(g/d)*	15.9 ± 1.6	14.7 ± 1.6	14.2 ± 1.7	12.5 ± 1.6	14.7 ± 1.7	17.6 ± 1.7*

Data are mean ± SD or mean ± SE

*P < 0.05. Analysis of variance or analysis of covariance with adjustment for total energy intakes was used

The risk of insulin resistance (odds and 95 % CI) in each tertile category of DII are shown in Table 3. After 3-years of follow-up, we found a significant positive association between DII and the risk insulin resistance in the highest compared to the lowest tertile, in the age- and sex-adjusted models (OR = 1.73, 95 % CI = 1.06–2.82, *P* for trend = 0.02); a borderline positive association was also observed between DII and the risk of insulin resistance in the fully adjusted model (OR = 1.66, 95 % CI = 0.96–2.86, *P* for trend = 0.06).

**Table 3:** Odds (95 % confidence interval) of the insulin resistance across tertile categories of dietary insulin index

	Dietary insulin index			
	Tertile1	Tertile2	Tertile3	*P* for trend
	< 81.6	81.6–86.3	≥86.3	
Model 1	1	1.43 (0.86–2.37)	1.73 (1.06–2.82)	0.02
Model 2	1	1.44 (.082–2.54)	1.66 (0.96–2.86)	0.07
Model 3	1	1.45 (0.82–2.56)	1.66 (0.96–2.86)	0.06

Model 1: adjusted for age and gender

Model 2: additional adjustment for body mass index, smoking, anti-diabetic medications, and physical activity

Model 3: additional adjustment for total energy intakes

The risk of insulin resistance (odds and 95 % CI) in each tertile category of DIL are presented in Table 4. After adjustment of all potential confounders, highest compared to the lowest tertile of DIL was significantly associated with increased risk of insulin resistance (odds ratio = 1.69, 95 % confidence interval = 1.01–2.89, *P* for trend = 0.06).

**Table 4:** Odds and 95 % Confidence Interval for Occurrence of the insulin resistance in each tertile categories of insulin load

	Dietary insulin load			
	Tertile1	Tertile2	Tertile3>	*P* for trend
	<794	794–1097	≥1097	
Model 1	1	1.07 (0.64–1.80)	1.90 (1.17–3.09)	0.06
Model 2	1	0.87 (0.49–1.56)	1.75 (1.05–2.96)	0.03
Model 3	1	0.81 (0.44–1.48)	1.69 (1.01–2.89)	0.04

Model 1: adjusted for age and gender

Model 2: additional adjustment for body mass index, smoking, anti-diabetic medications, and physical activity

Model 3: additional adjustment for total energy intakes

## Discussion

In this prospective study of healthy men and women, in multivariate adjusted model, DII had a borderline positive association with insulin resistance and increased DIL was accompanied with increased risk of insulin resistance after a 3-year of follow-up; these observed associations were independent of potential risk factors. In our study consumption of white rice and breads had a higher contribution in DIL; we also observed a significant negative correlation between DII and dietary 7intakes of fruits (*r* = −0.11, *P* < 0.01) vegetable (*r* = −0.14, *P* < 0.01), whole grains (*r* = −0.08, *P* < 0.01); moreover, DII was negatively correlated with total fat (*r* = −0.14, *P* < 0.01) and protein (*r* = −0.08, *P* < 0.01) and positively correlated with dietary carbohydrate (*r* = 0.17, *P* < 0.01).

In our study, mean DII and DIL was 84.0 ± 6.3 and 937 ± 254, respectively, and the top 10 FFQ items that contributed to DIL was white rice (24.8 %), traditional Iranian bread (21.3 %), white bread (13.5 %), potato (12.0 %), yogurt (11.2 %), baguette bread (2.5 %), banana (1.8 %), apple (1.4 %), ice cream (1.3 %) and orange (1.2 %). In the study of participants in Nurses’ Health Study and the Health Professionals Follow-Up Study, median DII and DIL was 41.7 and 840 in men, and 42.8 and 677 in women, respectively; in this population mashed potatoes, skimmed milk, cold cereal and dark bread had higher contribution to DIL [9, 13, 15]. Based on our finding insulinogenic foods can put individuals in higher risk of insulin resistance. Food pattern that restricted for insulinogenic foods can play an important role in prevention of insulin resistance. Consumption of rice and bread that are most insulinogenic foods, in Iranian population is high, so these food items can be responsible to explain the relationship between DII and DIL with insulin resistance in our population. A study in European outpatients with type 1 diabetes exhibit that bread and pasta make the largest contribution to the overall dietary GI [24].

To our knowledge, this is the first try to investigate the association of DII and DIL assessed by FFQs and insulin resistance. Previous studies have shown that glycemic index and glycemic load had a considerable effect on insulin response and a low- compared to high-glycemic index diet could improve insulin sensitivity [25, 26]. Dietary insulin index could directly quantifies the insulin secretion in response to the foods; it is suggested that in epidemiologic studies, DII may be more suitable than dietary glycemic index and glycemic load, or total carbohydrate intake to examine hypotheses the association of insulin exposure with the development of non-communicable chronic diseases [9]. Although carbohydrate is the primary stimulus for insulin secretion, protein-rich foods also elicit a significant insulin response and, when combined with carbohydrate, act synergistically to raise insulin concentrations and reduce glycemia. Also, addition of fat to a carbohydrate rich meal reduces postprandial glycemia but not the insulin response [10].

Previous investigations mainly evaluated the effects of DIL and DII on the risk of cancer including pancreatic, colorectal and endometrial cancer [13–15], and the association of DIL and DII with glucose and insulin homeostasis, and related metabolic disorders have been less evaluated the. The association of DIL and DDI with biomarkers of glycemic control, plasma lipids, inflammatory markers and body composition has been investigated in two studies [9, 27]. Nimptsch and colleagues in a study of Nurses’ Health Study and the Health Professionals Follow-Up Study, found that DII and DIL were not associated with plasma C-peptide, glycated hemoglobin, low-density lipoprotein cholesterol, C reactive protein, or interlukine-6 but a significant association was observed between DIL and DII with triglyceride concentrations and high-density lipoprotein levels [9]. The authors was also reported that, DIL and DII had a unexpected association with serum levels of insulin-like growth factor-binding protein 1 (IGFBP-1); a lower C-peptide concentration was observed in subjects who consumed a high DIL/DII which can be linked to β cell dysfunction [9]. In a prospective study, Joslowski et al. reported that a higher DII and DIL during puberty were associated with higher levels of percentage of body fat in young adulthood [27]. It is suggested that a high DIL and DII over a longer term may reduce insulin sensitivity, decrease lypolysis and also promote the development of body fat; high DIL and DII may also contribute to a higher body fat through cross-stimulation of both insulin and insulin growth factor-1 (IGF-1) secretion, and subsequently proliferation of preadipocytes, which may therefore contribute to body-fat formation [27].

The strengths of this study were a prospective setting, and use of a validated FFQ to assess regular dietary intake. Lack of data on postprandial levels of glucose and insulin to calculate the disposition index to accurate justifying β-cell function, was a limitation of this study; moreover, DII conceptually was calculated based on postprandial insulin response while fasting insulin, assessed in this study, reflected the basal insulin secretion.

## Conclusions

In conclusion, our finding demonstrated that a diet with a higher DII and DIL were associated with a higher risk of insulin resistance, findings which could imply the undesirable effects of a high-carbohydrate diet in the development of insulin resistance and its consequent metabolic disorders.

## Abbreviations

BMI: Body mass index
DII: Dietary insulin index
DIL: Dietary insulin load
ECLIA: Electrochemiluminescence immunoasaay
FCT: Food composition table
FFQ: Food frequency questionnaire
GI: Glycemic index
HOMA-IR: Homeostatic model assessment of insulin resistance
MET: Metabolic equivalent
TLGS: Tehran lipid and glucose study
WC: Waist circumference
